# Writing case reports for *ecancer*

**DOI:** 10.3332/ecancer.2015.ed49

**Published:** 2015-06-30

**Authors:** Priya Ranganathan, Sandeep B Bavdekar, C S Pramesh

**Affiliations:** 1 Department of Anaesthesiology, Tata Memorial Centre, Mumbai 400012, India; 2 Department of Paediatrics, TN Medical College and BYL Nair Hospital, Mumbai 400008, India; 3 Division of Thoracic Surgery, Tata Memorial Centre, Mumbai 400012, India

A case report is a *‘detailed report of the diagnosis, treatment, and follow-up of an individual patient’* [1]. Case reports have been a part of biomedical literature since ancient Egyptian times [2]. However, several misconceptions exist about case reports amongst physicians, journal readers and sometimes, even editors. In this editorial, we explain the definite value that case reports bring to medical progress and debunk some of the myths that surround them.

Case reports have been criticized on several fronts. They represent ‘extreme’ sensational findings which cannot be generalized to all patients. They function more as interesting anecdotes and less as actual scientific evidence. In this age of evidence-based medicine, case reports and case series have been described as *‘the lowest form of intellectual life’* and a leading anaesthesia journal actually carried an editorial on whether case reports should be *‘confined to the dustbin’* [3, 4]! Because of their low propensity to be cited, several journals have stopped accepting case reports for publication [5]. However, case reports undoubtedly add genuine value to medical literature. They serve as a platform to report new diseases, treatments and unexpected side effects which are yet to be published or widely known. For example, the world’s first heart transplant carried out by Christiaan Barnard in 1967 was publicized in a case report [6]. Case reports often provide the first possibility of an association or link, and form the basis for future structured research, as shown by initial publications documenting the relationship between thalidomide and phocomelia [7, 8]. In the field of cancer, the first descriptions of Hodgkin’s lymphoma and Burkitt’s lymphoma and the earliest hint of an association between chronic myeloid leukemia and the Philadelphia chromosome were documented as case reports [2].

Clinical trials usually accrue patients who are free from major co-morbidities; the extensive exclusion criteria of these trials reduce the generalizability of their results to the general population. Data related to patients who are not ‘fit’ for medical research can only be obtained from case reports and case series (while case reports deal with findings from individual patients, an extension of the case report is the ‘case series’ which deals with data from a group of patients). The creation of a case report is based on readily available data and needs very little time and resources to be invested – an ideal starting point for the budding researcher. For readers who are stymied by the intricacies of complex research methodology and biostatistics, case reports provide a quick and easy read, while arousing interest and awareness in the subject. Growing recognition of the importance of case reports has led to the resurgence of journals devoted to publishing case reports (for e.g., BMJ Case Reports, Journal of Medical Case Reports, Case Reports in Oncology).

The simplicity of the structure of the case report is in contrast to other forms of medical writing such as clinical trial reports. Case reports generally have 5 sections: abstract, introduction, case presentation, discussion, and conclusions [9, 10]. The abstract is brief and usually unstructured and conveys the gist of the case with its clinical relevance. The introduction lays down the background of the case explaining the importance of the medical problem being reported. The case presentation should read like a story in chronological order. Patient anonymity should be maintained and irrelevant details should be excluded. Relevant images and figures should be included. The discussion should include a literature review and an explanation of the case findings in the context of other evidence. The conclusion is a brief summary along with important take-home messages that the author wishes to convey. Recently, the CARE (CAse REport) guidelines were introduced to improve the quality and standardization of reporting of case reports [11]. It is also important to consider ethical aspects of reporting of cases. Patient photographs and images should be devoid of identifying tags and should be partially masked so as to obscure the identity of the patient. Many journals now require written informed consent to be taken from patients for use of their data and images in case reports. Some journals may also ask for Institutional Ethics Committee approval prior to publishing case reports. It would probably be helpful if journal editors could come up with uniform requirements for consent and ethics approval for case reports and also issue guidelines for cases where patients or their heirs are not available for obtaining consent.

With such an uncomplicated structure, it should be relatively easy to publish case reports. However, there has been a decline in the number of case reports published in journals [4, 12]. So, what do editors (and readers!) look for in a case report? A case report should deal with a novel finding such as identification of a new disease, unusual presentation of known diseases, previously unreported complications of treatment, unrecognized associations, different management techniques, innovative use of a drug or device (obviously, the ‘innovation’ should neither be unscientific, nor unethical), errors or ethical dilemmas in medical practice [10]. The case should be interesting and relevant to the readers of the journal. It should be hypothesis generating which means that it should have the potential to translate into further definitive research. The usual reasons for rejection of submitted case reports are lack of originality (‘me too’ submissions), use of scientifically or ethically unacceptable methods or wrong choice of journal (subject does not interest the readership). However, probably the most common reason a case report is rejected is the misconception of authors that the ‘rarity’ of an occurrence warrants reporting. Most editors would be reluctant to accept a case report of ‘only the 46th such reported case in the world’ unless there was a clear message from the report – mere rarity of an event is not a good enough reason for publication unless of course, it was amongst the first reports of a clinically important event like the association of HIV with Kaposi’s sarcoma.

The key question the author of a potentially publishable case report should answer in the positive is ‘Will the average clinician learn something of value from this case report?’ The humble case report cannot compete with clinical trials or systematic reviews in the pyramid of evidence-based medicine. However, it contributes to science in its own way and has an important place in biomedical literature.

## Conflicts of interest

The authors have no conflicts of interest.
